# Host immunometabolic reprogramming in paediatric refractory mycoplasma pneumoniae pneumonia: cellular circuits, multi-omics signatures, and translational opportunities

**DOI:** 10.3389/fcimb.2026.1954451

**Published:** 2026-10-28

**Authors:** Jingjing Yang, Shengtao Cui, Wei Li, Jian Liu

**Affiliations:** 1 Diagnosis and Treatment Center for Children, Affiliated Hospital of Changchun University of Chinese Medicine, Changchun, Jilin, China; 2 Department of Pediatrics, The Third Clinical Hospital of Changchun University of Chinese Medicine, Changchun, Jilin, China

**Keywords:** immunometabolism, metabolic reprogramming, *Mycoplasma pneumoniae* pneumonia, precision medicine, refractory pneumonia

## Abstract

Paediatric refractory Mycoplasma pneumoniae pneumonia (RMPP) is a heterogeneous syndrome in which persistent microbial stimulation, dysregulated host immunity and evolving airway injury may coexist despite macrolide treatment. This review integrates clinical, experimental and multi-omics evidence to examine RMPP through an immunometabolic framework. Mycoplasma pneumoniae reprograms airway epithelial cells, macrophages and neutrophils by altering glycolysis, mitochondrial and redox signalling, autophagy and lipid and amino-acid metabolism. These responses can support early antimicrobial defence yet become maladaptive when inflammatory output is uncoupled from bacterial clearance. Epithelial ATP-dependent signalling, macrophage inflammasome activation, neutrophil aconitate decarboxylase 1 (ACOD1; historically termed immune-responsive gene 1 [IRG1])-itaconate activity and extracellular trap accumulation illustrate this context-dependent transition. Adaptive immune remodelling, compartment-specific Th17-regulatory T-cell imbalance and incomplete mucosal antibody protection may further sustain disease. Emerging metabolomic, lipidomic, microbiome and single-cell studies support biological heterogeneity but remain predominantly cross-sectional and cannot distinguish causal drivers from consequences of severe disease or treatment. Current mechanistic evidence is strongest for epithelial glycolysis and the neutrophil ACOD1-itaconate axis in experimental M. pneumoniae systems, whereas direct cell-resolved functional validation in paediatric RMPP remains limited, and ferroptosis and several proposed therapeutic targets remain provisional. We therefore present immunometabolic states as a hypothesis-generating framework rather than as validated endotypes. Future studies should determine whether dynamic combinations of pathogen burden, cell-specific metabolic function, inflammatory activity and anatomical injury reproducibly identify biological states that precede progression and predict differential treatment response. Longitudinal, cell-resolved studies integrating metabolic flux, pathogen trajectories, treatment exposure and anatomical evolution are required before these candidate states can support mechanism-informed clinical stratification.

## 1 Introduction

Mycoplasma pneumoniae commonly causes mild, self-limiting respiratory infection but can also produce rapidly progressive pneumonia, airway obstruction and extrapulmonary injury in children. Following several years of reduced circulation during the coronavirus disease 2019 pandemic, M. pneumoniae re-emerged across multiple regions from 2023 onwards, again highlighting its burden among school-aged children and adolescents (Waites et al., 2017; ESGMAC MAPS study group, 2025). The principal clinical challenge is refractory M. pneumoniae pneumonia (RMPP), in which fever, respiratory manifestations and radiological abnormalities continue to progress despite macrolide treatment. RMPP is associated with dense consolidation, mucus plugging, plastic bronchitis, pulmonary necrosis and persistent structural lung abnormalities (Tong et al., 2022).

Although macrolide-resistant strains are prevalent, particularly in East Asia, microbiological resistance and clinical refractoriness are not equivalent (Kim et al., 2022). Some children with resistant infection recover without severe injury, whereas others deteriorate despite receiving an active antimicrobial agent; refractory disease may also occur without a recognized resistance mutation. Moreover, current definitions rely mainly on persistent fever and radiographic progression after a variable duration of macrolide therapy (Tong et al., 2022; Wang YS. et al., 2024). They therefore identify treatment failure without distinguishing inadequate pathogen suppression, persistent pathogen-derived stimulation, dysregulated host inflammation or defective resolution. Increased lactate dehydrogenase, D-dimer and inflammatory cytokines, including IL-6, IL-10, IL-18 and interferon-γ, are associated with RMPP (Zhang et al., 2016; Tong et al., 2022), but these markers quantify inflammatory severity rather than explain why inflammation persists, bacterial clearance remains inefficient or airway injury progresses.

Immunometabolic reprogramming provides a framework for connecting these processes. Immune and structural cells reorganize glycolysis, mitochondrial respiration, tricarboxylic acid cycle activity, fatty-acid metabolism and amino-acid utilization to support cytokine production, redox regulation, antimicrobial activity and survival (O'Neill et al., 2016). These changes can be protective or pathological depending on their cellular context and duration. In bronchial epithelial cells, M. pneumoniae-induced aerobic glycolysis promotes ATP-dependent inflammatory signalling but is also required for bacterial elimination (He et al., 2024). In neutrophils, experimental activation of the aconitate decarboxylase 1 (ACOD1; historically termed immune-responsive gene 1 [IRG1])–itaconate pathway reduces mitochondrial reactive oxygen species, impairs bacterial killing and delays apoptosis (Wang C. et al., 2024). These observations illustrate that metabolic reprogramming can, in specific experimental contexts, contribute to the dissociation between inflammatory activity and antimicrobial efficiency (He et al., 2024; Wang C. et al., 2024). They should not, however, be interpreted as evidence that all children meeting clinical criteria for RMPP share a common metabolic mechanism, because persistent viable organisms, delayed effective antimicrobial therapy, pathogen-derived inflammatory stimulation, co-infection, airway obstruction and established tissue injury may contribute independently or in combination.

Systems-level studies further suggest that severe MPP comprises biologically distinct host-response states. Single-cell analysis of bronchoalveolar lavage fluid has identified coordinated remodelling of epithelial, myeloid and lymphoid compartments, including severity-associated macrophage and neutrophil programmes and disrupted lymphocyte responses (Shen et al., 2024). Plasma multi-omics has also demonstrated activation of the kynurenine pathway and suppression of indole-related metabolites in association with inflammatory severity (Tang et al., 2025). These findings support biological heterogeneity and provide a rationale for testing endotype-based hypotheses, but they do not yet establish discrete immunometabolic endotypes in RMPP. Most paediatric studies remain cross-sectional, apply inconsistent definitions of refractoriness and analyse samples obtained after antimicrobial or immunomodulatory treatment. Moreover, direct metabolic characterization of defined pulmonary cell populations is scarce. Metabolic signatures detected in blood, bulk bronchoalveolar lavage fluid, experimental models or unrelated infections may therefore reflect disease severity, treatment exposure or established tissue injury rather than mechanisms that initiated refractoriness.

This narrative review therefore examines how metabolic reprogramming of airway epithelial cells and innate and adaptive immune populations may connect pathogen persistence, ineffective defence and progressive lung injury in paediatric RMPP. It evaluates glycolysis, mitochondrial and tricarboxylic acid cycle remodelling, lipid and amino-acid metabolism, redox regulation and metabolically controlled cell death, while integrating emerging single-cell and multi-omics evidence and considering their implications for biomarkers and host-directed treatment. PubMed, Web of Science and Embase were searched from database inception to June 15, 2026, using combinations of terms related to M. pneumoniae, refractory pneumonia, children, immunometabolism, metabolic reprogramming, glycolysis, mitochondrial metabolism, itaconate, lipid metabolism, amino-acid metabolism, metabolomics, lipidomics and single-cell sequencing. Paediatric RMPP studies were prioritized, followed by paediatric MPP cohorts and experimental M. pneumoniae models; evidence from other pulmonary infections was included only when it addressed a defined mechanistic gap.

## 2 Paediatric RMPP: from a clinical label to a host-pathogen systems disorder

### 2.1 Refractoriness is a composite clinical endpoint

RMPP is not a discrete disease entity but a composite outcome describing pneumonia that continues to progress after treatment begins. Definitions generally combine persistent fever with radiological deterioration after several days of macrolide therapy, although treatment intervals vary and refractory, macrolide-unresponsive and severe MPP often describe overlapping populations (Tong et al., 2022; Wang YS. et al., 2024). Consequently, the same label may encompass delayed exposure to an active antibiotic, persistent inflammation despite microbiological control, or early necrotizing injury, each requiring a different therapeutic approach.

At the group level, RMPP cohorts show recurrent biological associations, including greater airway bacterial burden, increased neutrophil accumulation and higher concentrations of inflammatory cytokines than non-refractory MPP (Wang et al., 2014). Lower-airway M. pneumoniae DNA also correlates with neutrophil abundance (Wang et al., 2014). These observations indicate that microbial persistence and inflammatory amplification can coexist, but they do not demonstrate that all refractory cases share the same underlying mechanism. RMPP should therefore be regarded as a heterogeneous clinical endpoint in which the relative contributions of microbial persistence, ineffective host defence, inflammatory injury and anatomical obstruction may differ among patients and across disease stages.

Host background modifies this response. Older children are overrepresented in many cohorts, potentially reflecting stronger recall immunity rather than intrinsic airway susceptibility (Wang et al., 2014; Tong et al., 2022). Atopy and asthma have also been associated with refractoriness, although retrospective evidence cannot distinguish causal effects from differences in clinical investigation (Shin et al., 2014). Sex may represent another biological modifier of infection-related immunity; however, current epidemiological evidence does not demonstrate a consistent sex difference in paediatric RMPP. A recent meta-analysis including 28 studies and 6,374 children found no significant association between sex and refractory disease (Chen et al., 2025). This does not exclude sex-dependent differences in immune or metabolic responses, particularly across age and pubertal stages, but such interactions have not been adequately investigated in RMPP. Future cohorts should therefore report sex-stratified clinical and molecular data and formally examine interactions among sex, age, immune phenotype and treatment response rather than treating sex as an established risk factor. RMPP can thus be classified along three intersecting dimensions: microbiological persistence, shaped by antimicrobial susceptibility, treatment timing and pathogen burden; immune dysregulation, reflected in epithelial, myeloid and lymphocyte responses; and anatomical progression, including consolidation, mucus plugging, obstruction and necrosis. Because these dimensions need not evolve together, heterogeneous trajectories can converge on the same clinical endpoint.

### 2.2 Pathogen sensing establishes a metabolically demanding inflammatory state

Prolonged attachment of M. pneumoniae to the respiratory epithelium provides continuous exposure to adhesins, lipoproteins, membrane components and secreted virulence factors. Among them, the community-acquired respiratory distress syndrome (CARDS) toxin is an ADP-ribosylating and vacuolating cytotoxin that alters host-cell function and damages the epithelium (Kannan and Baseman, 2006). Recombinant toxin alone can induce airway inflammation, mucus abnormalities, and eosinophilic and lymphocytic infiltration (Medina et al., 2012), showing that pulmonary pathology can persist independently of bacterial replication.

M. pneumoniae itself is also biologically heterogeneous. Experimental strains differ in CARDS toxin production, airway colonization, persistence and the severity of pulmonary inflammation, indicating that pathogen-intrinsic properties can modify the magnitude and duration of host stimulation (Techasaensiri et al., 2010). Comparative genomic, transcriptomic and proteomic analyses of clinical isolates have identified variation in P1-associated loci, glycerol metabolism, peroxide production and strain-dependent CARDS toxin expression (Lluch-Senar et al., 2015). However, available comparative genomic studies have not identified a single virulence genotype that consistently explains severe or refractory clinical phenotypes (Jiang et al., 2024). Thus, variation in adhesion, toxin expression, metabolic activity and antimicrobial resistance may modify host immunometabolic responses, but the available evidence does not support a discrete “hypervirulent RMPP clone”. Future host-response studies should therefore incorporate pathogen genotype, resistance determinants and, where feasible, quantitative virulence-factor expression.

CARDS toxin also ADP-ribosylates NOD-like receptor protein 3 (NLRP3), promoting inflammasome activation and IL-1β processing (Bose et al., 2014). Because NLRP3 responds to extracellular ATP, potassium efflux, mitochondrial dysfunction and reactive oxygen species, toxin-induced injury can reinforce a circuit linking metabolic stress to inflammation. In parallel, membrane lipoproteins activate Toll-like receptor 2 signalling. Pulmonary macrophage depletion or disruption of MyD88-NF-κB signalling impairs early bacterial control (Lai et al., 2010), confirming that resident macrophages are essential for defence but also drive cytokine and chemokine production. Persistent signalling may therefore maintain macrophages and epithelial cells in a metabolically demanding inflammatory state whose antimicrobial benefit progressively declines.

These pathogen signals are partly non-redundant. TLR2-dependent IL-1α and IL-12 p40 production recruits neutrophils, whereas CARDS toxin can induce neutrophil influx independently of TLR2 (Tamiya et al., 2021). Such parallel pathways explain why suppressing bacterial replication may not immediately terminate inflammation: residual toxin, damage-associated signals and self-amplifying cytokine circuits can remain active after the initiating microbiological event has diminished.

### 2.3 Refractoriness emerges when inflammation is uncoupled from clearance

Neutrophils illustrate how vigorous immunity can become inefficient. In children with MPP, airway IL-8 is accompanied by increased myeloperoxidase, neutrophil elastase and matrix metalloproteinase 9, which correlate with fever duration and hospitalization (Chen et al., 2016). Although neutrophils restrict infection through phagocytosis, oxidative killing and extracellular traps, the same mechanisms disrupt epithelial barriers, increase vascular permeability and remodel the extracellular matrix. Once recruitment exceeds effective bacterial killing, additional neutrophils may intensify injury without improving clearance.

Experimental findings support this dissociation. In some mouse models, neutrophil depletion reduces lung injury without substantially impairing bacterial elimination, while TLR2-dependent macrophage cytokines and CARDS toxin independently sustain neutrophil recruitment (Tamiya et al., 2021). Thus, neutrophils may be protective early but predominantly injurious when recruitment persists in a metabolically stressed airway. M. pneumoniae further shifts this balance through the secreted nuclease Mpn491, which degrades the DNA scaffold of neutrophil extracellular traps and protects the organism from NET-mediated killing (Yamamoto et al., 2017). The host consequently retains the tissue-damaging effects of NETosis while losing part of its antimicrobial benefit, explaining how intense neutrophilic inflammation can coexist with persistent bacterial DNA.

Comparable inefficiency may develop in other compartments: macrophages can remain cytokine-producing yet lose bacterial control, epithelial cells can amplify purinergic and inflammasome signalling while losing barrier integrity, and lymphocytes can sustain cytokine production without promoting resolution. Hypoxia, nutrient depletion, extracellular lactate, mitochondrial stress and oxidant accumulation further reduce the efficiency with which inflammation is converted into clearance.

This distinction also guides treatment. Corticosteroid responsiveness indicates that host inflammation can become a dominant disease driver, but immunosuppression may be less effective or counterproductive when bacterial burden remains high. Conversely, changing antibiotics addresses microbiological persistence but cannot directly reverse mucus plugging, inflammasome activation or established injury. Combined antimicrobial and host-directed treatment should therefore target the rate-limiting component of the pathogen-host circuit rather than clinical severity alone.

The clinical RMPP phenotype may arise from one or more interacting processes, including incomplete removal of microbial stimuli, inefficient coupling of immune activation to antimicrobial killing, failure to terminate inflammation once its protective value declines, and progression of established anatomical injury. Immunometabolic reprogramming represents one potential modifier of these processes by influencing cellular energetic capacity, effector function and survival, but its contribution is likely to vary according to pathogen burden, disease stage, treatment exposure and the affected cellular compartment. The following sections therefore examine candidate cell-specific metabolic mechanisms rather than assuming a uniform metabolic basis for refractoriness. This hypothesis-generating framework is summarized in **Figure 1**.

**Figure 1:**
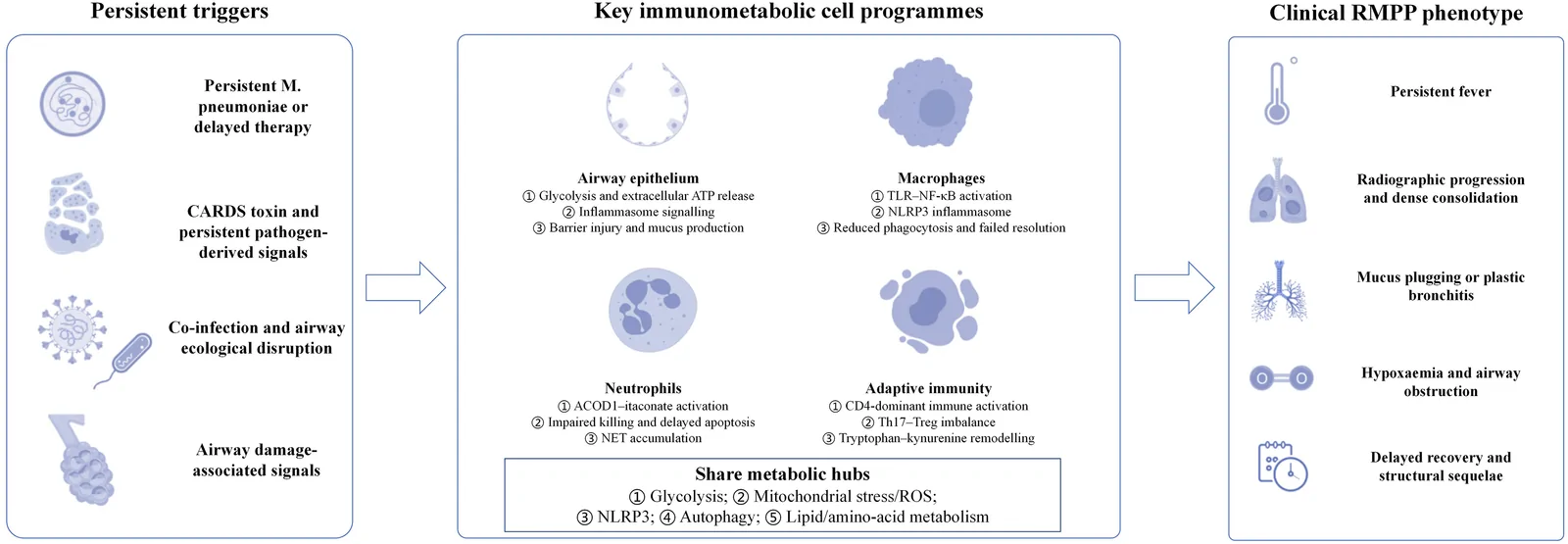
Simplified immunometabolic framework of paediatric refractory Mycoplasma pneumoniae pneumonia. Arrows denote proposed and context-dependent relationships rather than an established linear causal sequence; the relative contributions of microbial persistence, host-response programmes and anatomical injury may differ among patients and across disease stages.

## 3 Metabolic reprogramming of airway and innate immune cells

### 3.1 The airway epithelium: host defence at a metabolic cost

The respiratory epithelium is not merely the surface to which M. pneumoniae attaches. It must maintain barrier integrity and mucociliary clearance while generating antimicrobial and inflammatory signals. Infection therefore creates competition between energy-intensive defence and tissue homeostasis.

This trade-off is illustrated by epithelial glycolysis. M. pneumoniae increases glucose uptake and aerobic glycolysis in bronchial epithelial cells, promoting intracellular ATP production, extracellular ATP release, purinergic signalling, inflammatory cytokine production and bacterial elimination (He et al., 2024). Glycolysis consequently supports early defence while amplifying IL-1β and IL-18 signalling. Its effect depends less on whether it is activated than on whether its magnitude and duration remain coupled to bacterial burden.

Persistent glycolysis may also reshape the airway microenvironment. Increased glucose consumption, lactate production and extracellular acidification could create nutrient gradients within poorly ventilated consolidation, influencing ciliary activity, proteases and infiltrating immune cells, although spatial metabolomic evidence in RMPP remains unavailable. An important limitation is that the available epithelial evidence derives largely from conventional *in vitro* systems maintained under relatively normoxic, glucose-replete conditions, which do not reproduce the spatial metabolic constraints of severe RMPP lungs. Consolidated, poorly ventilated and mucus-obstructed regions are likely to differ substantially in oxygen availability, substrate delivery, extracellular pH and metabolite clearance. It is therefore unknown whether epithelial cells within advanced lesions sustain the glycolytic phenotype observed under standard culture conditions or instead develop progressive energetic stress, mitochondrial dysfunction or cell death. Direct spatial or cell-resolved metabolic measurements from affected paediatric airways are currently unavailable, and the *in vitro* glycolytic phenotype should not be assumed to represent all anatomical regions or disease stages of RMPP. Meanwhile, infection induces reactive oxygen species, alters mitochondrial and oxidative-stress proteins and causes DNA double-strand damage in lung epithelial cells (Sun et al., 2008). Epithelial injury therefore reflects both infection-derived oxidants and oxidants released by recruited neutrophils.

The ability to compensate for this stress may be as important as its magnitude. Environmental tobacco smoke suppresses the adaptive pulmonary glutathione response to M. pneumoniae, increasing oxidative injury (Kariya et al., 2008). Although not demonstrated specifically in paediatric RMPP, this finding shows that environmental exposure, nutrition, pre-existing airway inflammation and inherited antioxidant capacity may modify metabolic reserve without directly altering pathogen recognition. Autophagy adds further complexity: M. pneumoniae cytadherence activates autophagy-dependent inflammatory signalling through Toll-like receptor 4 even without canonical Toll-like receptor 2 signalling (Shimizu et al., 2014). Effective autophagic flux may remove damaged mitochondria, whereas incomplete or prolonged activation may sustain nuclear factor-κB and inflammasome signalling.

These pathways converge on epithelial fitness. Glycolysis supplies energy, oxidants signal stress and autophagy determines whether damaged components are recycled or retained as inflammatory stimuli. If appropriately resolved, they support bacterial control and barrier preservation; if infection persists, the epithelium becomes a continuing source of extracellular ATP, cytokines, oxidants and damage-associated signals. Epithelial metabolic responses may therefore contribute to the transition from localized infection to self-sustaining airway inflammation, although their relative importance is likely to vary across anatomical regions and disease stages.

### 3.2 Macrophages: metabolic control of sensing, killing and resolution

Resident and recruited macrophages interpret the airway environment and determine whether it supports clearance, leukocyte recruitment or repair. Their states cannot be adequately represented by the binary M1-M2 framework because inflammatory, antimicrobial, regulatory and remodelling programmes can coexist and are shaped by local substrates and signals. Early activation is protective: pulmonary macrophage depletion or disruption of MyD88-nuclear factor-κB signalling impairs control of M. pneumoniae (Lai et al., 2010). However, the same pathway produces cytokines and chemokines, creating a trade-off between insufficient activation and persistent inflammation.

The NLRP3 inflammasome lies near this transition. In addition to direct NLRP3 modification by CARDS toxin (Bose et al., 2014), M. pneumoniae-derived lipids activate Toll-like receptor 4 and reinforce interactions among nuclear factor-κB, autophagy and inflammasome signalling (Luo et al., 2021). These processes are metabolically linked: nuclear factor-κB supports cytokine biosynthesis, autophagy regulates substrates and damaged mitochondria, and NLRP3 responds to extracellular ATP, ion flux, oxidants and mitochondrial dysfunction. Persistent activation may therefore reflect failure to restore macrophage metabolic homeostasis rather than continued receptor stimulation alone.

Because IL-1β and IL-18 can support early defence and shape lymphocyte responses, complete inflammasome suppression might impair pathogen control. Pathology is more likely to arise when cytokine production persists after phagocytosis, oxidative killing or efferocytosis has declined. Macrophages may then sustain inflammation without efficiently removing bacteria or apoptotic neutrophils.

Macrophage sensing also extends beyond established Toll-like receptor pathways. M. pneumoniae proteins have been reported to interact with nucleotide-binding oligomerization domain-containing protein 2 and activate macrophage nuclear factor-κB signalling (Wang Y. et al., 2024). Because the organism lacks the conventional peptidoglycan ligand for this receptor, the finding suggests non-canonical pathogen recognition. Although its relevance to human RMPP remains uncertain, it illustrates how multiple upstream signals can converge on shared metabolic and transcriptional nodes.

Extracellular metabolites can further modify macrophage behaviour. In M. pneumoniae-stimulated THP-1 cells, acetate, propionate and butyrate reduced reactive oxygen species, NLRP3 activation and pro-inflammatory cytokines while increasing IL-10-related responses (Wen et al., 2021). This supports metabolic regulation of macrophage activation but does not establish short-chain fatty acids as an RMPP treatment. THP-1 cells do not reproduce pulmonary macrophage diversity, airway concentrations in affected children are unknown, and effects may vary by concentration and cell type.

A clinically important possibility is that RMPP macrophages fail to transition from inflammatory defence to resolution. Efferocytosis, debris removal, epithelial restoration and matrix repair require mitochondrial and lipid programmes that may be inhibited by hypoxia, lactate, oxidants and persistent pathogen signals. Macrophages could consequently remain cytokine-producing yet ineffective at restoring homeostasis. Severity-associated macrophage subsets have been identified by single-cell transcriptomics (Shen et al., 2024), but transcription does not establish metabolic flux. Direct measurements of glycolysis, mitochondrial respiration, substrate use and efferocytosis in bronchoalveolar macrophages are needed before these states can be described as polarization or metabolic exhaustion.

### 3.3 Neutrophils: when metabolic persistence exceeds antimicrobial return

Among the pathways discussed here, neutrophil metabolism provides one of the strongest experimental links between M. pneumoniae infection and altered antimicrobial function, although direct functional validation in paediatric RMPP remains limited. They accumulate in severe disease, correlate with pathogen burden and release proteases, oxidants and extracellular traps (Wang et al., 2014; Chen et al., 2016), yet greater abundance does not necessarily produce greater antimicrobial activity. Neutrophils depend mainly on glycolysis, allowing function in hypoxic tissue, while glucose-derived pentose phosphate pathway activity supplies NADPH for oxidative killing. Redistribution of carbon or disruption of redox signalling may therefore preserve viability, migration and inflammatory output while impairing bacterial killing.

The ACOD1-itaconate pathway provides experimental evidence for one form of this functional uncoupling. Wang et al. found that bronchoalveolar lavage fluid itaconate concentrations correlated positively with neutrophil abundance in patients with severe M. pneumoniae pneumonia and detected increased ACOD1 (IRG1) expression in human airway neutrophils (Wang C. et al., 2024). However, the functional evidence linking increased neutrophil itaconate to suppression of mitochondrial reactive oxygen species, impaired bacterial killing and delayed apoptosis was derived predominantly from mouse loss-of-function and adoptive-transfer experiments (Wang C. et al., 2024). Potential species differences in ACOD1 induction, intracellular itaconate abundance and downstream redox responses further limit quantitative extrapolation from murine neutrophils to human pulmonary neutrophils. Direct demonstration of the same metabolic-functional relationship in purified pulmonary neutrophils from children with RMPP is therefore lacking. Future studies should quantify intracellular itaconate and ACOD1 activity in sorted blood and bronchoalveolar neutrophils and relate these measurements to mitochondrial reactive oxygen species, oxidative burst, ex vivo antimicrobial activity, apoptosis and serial pathogen burden. Importantly, the biological effect of itaconate is cell-context dependent. In human macrophages, endogenous ACOD1-derived itaconate restrains innate cytokine production, whereas experimental M. pneumoniae infection suggests that increased neutrophil itaconate can compromise antimicrobial efficiency (Bourner et al., 2024; Wang C. et al., 2024). Thus, a metabolite that appears maladaptive in one cellular compartment may remain protective in another, arguing against systemic ACOD1 inhibition without cell-specific validation.

Age may influence this state. Increasing age and peripheral neutrophil abundance were independently associated with RMPP, while neutrophils from older children showed greater reactive oxygen species generation, myeloperoxidase release and extracellular trap formation, together with changes in apoptosis, NADPH pathways, mitochondrial function and oxidative stress (Zhao et al., 2021). These findings may explain the predominance of RMPP in school-aged children, although the small mechanistic subgroup and association between age and phenotype prevent causal conclusions.

NETosis provides a tissue-level manifestation of neutrophil inefficiency. M. pneumoniae induces neutrophil extracellular traps but can degrade their DNA scaffold through the secreted nuclease Mpn491 (Yamamoto et al., 2017). The organism thereby escapes part of the antimicrobial effect while extracellular histones, myeloperoxidase and elastase remain in the airway. NETs may also activate NLRP3 signalling, connecting neutrophil death with macrophage inflammasome activity. Circulating and airway NET markers are increased in paediatric MPP and are associated with NLRP3 activation and disease severity (Yang et al., 2024). NET products can therefore activate macrophages and injure epithelial cells, which release further neutrophil-recruiting signals.

This circuit is particularly evident in plastic bronchitis. Children with MPP-associated plastic bronchitis show extensive neutrophil infiltration and NET formation in blood, bronchoalveolar lavage fluid and bronchial casts, with neutrophil and NET abundance correlating with clinical outcomes (Huang et al., 2025). Extracellular DNA increases mucus viscosity, while proteases and epithelial injury promote mucin release and barrier disruption. Airway casts may thus represent inflammation physically embedded in the airway after neutrophil activity exceeds local clearance capacity.

NETs are not uniformly pathogenic, because controlled early formation may restrict microbial dissemination. The important distinction is between transient NET formation and sustained accumulation in the presence of bacterial nuclease activity and impaired mucociliary clearance. NET measurements should therefore be interpreted alongside pathogen burden, nuclease activity, neutrophil viability and airway clearance rather than as isolated circulating markers.

Together, epithelial cells, macrophages and neutrophils form a metabolically coupled network. The epithelium establishes airway energetic and redox conditions, macrophages integrate microbial and damage signals, and neutrophils provide rapid defence at substantial tissue cost. RMPP may develop when these functions become temporally misaligned: epithelial glycolysis and macrophage inflammation persist, neutrophils survive despite inefficient killing, and autophagy, antioxidant defence, efferocytosis and mucociliary clearance fail to restore homeostasis. The available evidence is therefore consistent with the hypothesis that loss of temporal and functional coordination among structural and innate immune compartments may contribute to refractory progression in a subset of patients, although it has not been established as a universal or defining pathological event in RMPP.

## 4 Adaptive immune dysregulation through a metabolic lens

### 4.1 T-cell activation does not necessarily confer effective protection

The predominance of RMPP in school-aged children is often attributed to stronger adaptive immunity, but greater T-cell activation does not necessarily produce more effective pathogen control. Antigen-experienced T cells may accelerate clearance while also amplifying macrophage activation, neutrophil recruitment and epithelial injury. Protection therefore depends on whether T-cell differentiation, localization and metabolism remain aligned with bacterial elimination. High-dimensional flow cytometry shows that children with MPP have expanded activated CD4^+^ T cells but reduced total and central-memory CD8^+^ populations compared with children with non-MPP pneumonia. Severe disease is particularly associated with activated and central-memory CD4^+^ cells rather than uniform T-cell expansion (Li et al., 2024). However, reduced circulating CD8^+^ cells may reflect tissue recruitment, activation-induced contraction or functional impairment, so peripheral counts do not directly represent pulmonary immune competence.

The antigen-specific response also appears predominantly CD4^+^. M. pneumoniae antigens induce interferon-γ-producing CD4^+^ effector-memory cells, whereas corresponding CD8^+^ responses are limited. The magnitude of this response correlates with radiographic involvement and persists during convalescence (Pánisová et al., 2021). It may support early macrophage-mediated clearance but sustain myeloid inflammation after its antimicrobial benefit declines. Consistent with this possibility, bronchoalveolar T-cell cytokine profiles in children who develop M. pneumoniae-associated bronchiolitis obliterans are associated with later pulmonary-function abnormalities (Xu et al., 2020). Although these observations do not establish that T cells initiate structural injury, they show that local adaptive activity can persist beyond uncomplicated infection.

Metabolic state may explain this dissociation between activation and protection. Naive and memory T cells primarily use mitochondrial oxidative metabolism, whereas effector differentiation requires greater glucose uptake, glycolysis, amino-acid utilization and biosynthesis (O'Neill et al., 2016). This transition enables proliferation and cytokine production but creates dependence on local nutrients. Hypoxic, glucose-limited and lactate- and oxidant-rich consolidated airways may preserve inflammatory cytokine production while impairing proliferation, cytotoxicity or memory formation.

Current evidence does not justify describing this state as T-cell exhaustion. Reduced CD8^+^ numbers and prolonged antigen exposure are compatible with exhaustion, but inhibitory receptors, mitochondrial reserve, polyfunctional cytokine production and antigen-specific killing have rarely been examined together in paediatric RMPP. Corticosteroids further confound lymphocyte trafficking and metabolism. The more defensible interpretation is a qualitative redistribution of adaptive immunity, with prominent CD4^+^ inflammation but less consistent CD8^+^ effector and memory coordination.

This imbalance may also be compartment-specific. Blood contains circulating cells, whereas bronchoalveolar lavage samples the airway lumen rather than consolidated parenchyma or lymphoid tissue. Reduced circulating lymphocytes may therefore coexist with intense local activation. Paired blood and pulmonary sampling, together with separation of antigen-specific from bystander cells, will be necessary to localize adaptive dysfunction.

### 4.2 The Th17-Treg axis is dynamic, compartment-specific and metabolically regulated

The Th17-regulatory T-cell axis is often reduced to a simple model in which increased Th17 and reduced Treg activity drive RMPP. Although IL-17 promotes neutrophil recruitment and Tregs restrain inflammation, current evidence does not support a fixed relationship between IL-17 abundance and severity. Peripheral blood studies report increased Th17 cells, reduced Treg frequencies and a higher Th17-to-Treg ratio in RMPP than in non-refractory disease (Guo et al., 2016). Circulating Th17 expansion and increased IL-17 have also been observed in paediatric MPP (Wang et al., 2016), supporting a pathway through which prolonged Th17 activity reinforces epithelial chemokine production, neutrophil recruitment and delayed resolution.

This response is linked to innate sensing. CD16^+^ monocytes from children with MPP produce more IL-23, while CARDS toxin induces IL-23 through Toll-like receptor 4. IL-23 correlates with IL-17, and both conventional Th17 and IL-17-producing γδ T cells are expanded (Wang et al., 2020). Monocytes may therefore stabilize inflammatory lymphocyte programmes rather than simply recruit them.

Airway findings complicate this model. In two paediatric cohorts, bronchoalveolar IL17A messenger RNA and protein were lower in severe and refractory MPP than in mild disease and were also reduced in children with pleural effusion (Yang et al., 2017). Increased peripheral Th17 activity and reduced airway IL-17A may reflect different compartments or disease stages. Local IL-17 may support early mucosal defence and bacterial clearance, whereas prolonged systemic Th17 activation may continue after protective airway signalling becomes ineffective. Differences in producing cell type, sampling time, corticosteroid exposure, consolidation and recovery of viable airway cells could further explain the discrepancy. The Th17-Treg ratio is therefore a spatially and temporally variable state rather than a stable patient characteristic.

Co-stimulatory signals provide additional regulation. Reduced miR-29c expression in children with MPP has been associated with increased B7-H3 expression and enhanced Th17 responses (Li et al., 2019). This pathway may bias T-cell differentiation during persistent stimulation, but its specificity to RMPP and its effect on pathogen clearance remain unproven. Metabolic requirements offer a plausible basis for these shifts. Th17 differentiation is promoted by mTOR- and hypoxia-inducible factor 1α-dependent glycolysis, whereas glycolytic inhibition suppresses Th17 development and favours FOXP3^+^ Treg differentiation (Shi et al., 2011). Effector CD4^+^ cells generally have greater glycolytic activity, while Tregs depend more on mitochondrial and lipid oxidative metabolism (Michalek et al., 2011). These findings derive primarily from experimental systems, but they are relevant to the hypoxic and inflammatory conditions of pneumonia.

Early airway hypoxia may stabilize hypoxia-inducible factor 1α and favour glycolytic Th17 programmes, while oxidative stress and mitochondrial damage impair Treg stability. Prolonged nutrient depletion may eventually compromise both populations, producing low airway IL-17 despite unresolved inflammation. Thus, high circulating IL-17 may indicate inflammatory amplification, whereas low bronchoalveolar IL-17 may reflect impaired mucosal defence rather than resolution. Therapeutic interpretation requires paired assessment of cell frequency, cytokine production, regulatory function, anatomical compartment and bacterial burden.

### 4.3 Humoral immunity: vigorous antibody production but incomplete mucosal clearance

The B-cell response to M. pneumoniae is robust, but serum IgM and IgG do not distinguish protective clearance from recent antigen exposure. This is particularly important in RMPP, where strong humoral activation can coexist with persistent airway bacterial DNA.

Acute childhood pneumonia induces a transient population of circulating M. pneumoniae-specific antibody-secreting cells. These cells appear early and decline more rapidly than serum antibodies or upper-airway bacterial DNA, making them potential indicators of active infection (Meyer Sauteur et al., 2020). P1-specific antibody-secreting cells are also readily detected, with IgM-producing cells dominating the acute response. Their frequency correlates with fever duration, while IgG-secreting-cell frequency correlates with serum antibody titres (Lai et al., 2022). A prolonged response may therefore indicate sustained antigen exposure, although it remains unclear whether it prevents refractory progression.

Humoral protection is anatomically restricted. B-cell-deficient mice show impaired pulmonary clearance, which is restored by pathogen-specific serum immunoglobulin. However, the same antibodies have little effect on persistent upper-airway carriage, where local IgA and compartment-specific B-cell responses appear more important (Meyer Sauteur et al., 2018). Systemic antibodies may therefore support lower-airway clearance without sterilizing the nasopharyngeal reservoir.

Antigen specificity is equally important. Antibodies against M. pneumoniae proteins contribute to defence, whereas antibodies against bacterial glycolipids are less clearly protective and may cross-react with neural glycolipids (Meyer Sauteur et al., 2019). This distinction provides a potential connection between infection and immune-mediated extrapulmonary manifestations. Total IgM or IgG titres remain biologically imprecise because antibody effects depend on antigen target, affinity, Fc function and tissue distribution.

B-cell activation also has substantial metabolic demands. Proliferation requires nucleotide synthesis, membrane production and mitochondrial activity, while plasmablasts depend on glucose and amino-acid metabolism for immunoglobulin synthesis and endoplasmic-reticulum expansion. Persistent antigen could therefore maintain short-lived, IgM-dominant plasmablast responses without generating equivalent high-affinity mucosal protection. However, paediatric RMPP studies have not yet integrated B-cell subsets, metabolic flux, antibody specificity and lower-airway clearance.

B cells may additionally present antigen, produce cytokines and regulate Th1 and Th17 differentiation, while immune complexes and complement could contribute to extrapulmonary vascular, neurological or dermatological injury. These possibilities remain insufficiently resolved, and serum immunoglobulin concentrations alone cannot determine whether B cells are protective, dysregulated or simply responding to persistent infection.

Adaptive immunity in RMPP is therefore best understood as a coordination failure. CD4^+^ effector-memory responses may support macrophage activation while prolonging injury; Th17 and Treg functions vary across compartments and disease stages; and strong antibody production may protect the lung without eliminating mucosal carriage. Metabolism connects these processes by controlling lymphocyte differentiation, effector function, longevity and memory. The central defect may be failure to convert antigen recognition into durable clearance followed by timely resolution. The principal cell-specific immunometabolic programmes and their evidentiary context are summarized in **Table 1**.

**Table 1:** Cell-specific immunometabolic programmes relevant to paediatric refractory Mycoplasma pneumoniae pneumonia.

Cell or compartment	Principal immunometabolic programme	Functional interpretation	Evidence context	Reference
Airway epithelium	① Increased glucose uptake and aerobic glycolysis; ② extracellular ATP release; ③ oxidative stress and autophagy-related signalling	Glycolysis supports cytokine production and bacterial elimination during early defence, whereas persistent signalling may promote inflammasome activation, barrier injury and impaired mucociliary function	Experimental *M. pneumoniae* epithelial models	(Kariya et al., 2008; Sun et al., 2008; Shimizu et al., 2014; He et al., 2024)
Macrophages	① TLR2/TLR4–MyD88–NF-κB signalling; ② NLRP3 inflammasome activation; ③ autophagy-related regulation	Macrophage activation supports early bacterial control and leukocyte recruitment, but persistent inflammasome signalling may sustain inflammation after its antimicrobial benefit declines	Experimental *M. pneumoniae* models and bronchoalveolar single-cell transcriptomics	(Lai et al., 2010; Bose et al., 2014; Luo et al., 2021; Shen et al., 2024)
Neutrophils	① Glycolytic metabolism; ② ACOD1–itaconate activation; ③ altered mitochondrial ROS; ④ NET formation	Itaconate suppresses mitochondrial ROS-dependent bacterial killing and delays apoptosis, while persistent NETs, oxidants and proteases contribute to airway injury and bronchial casts	Pediatric cohorts and experimental *M. pneumoniae* models	(Wang et al., 2014; Chen et al., 2016; Yamamoto et al., 2017; Zhao et al., 2021; Wang C. et al., 2024; Yang et al., 2024; Huang et al., 2025)
T lymphocytes	① CD4-dominant activation; ② compartment-dependent Th17–Treg imbalance; ③ differential glycolytic and oxidative metabolic requirements	Adaptive activation may support early antimicrobial defence but can also sustain myeloid recruitment and fail to generate coordinated regulatory or memory responses	Pediatric MPP/RMPP cohorts and mechanistic T-cell studies	(Michalek et al., 2011; Shi et al., 2011; Guo et al., 2016; Wang et al., 2016; Yang et al., 2017; Li et al., 2019; Wang et al., 2020; Xu et al., 2020; Pánisová et al., 2021; Li et al., 2024)
B cell and humoral immunity	① Acute antibody-secreting-cell responses; ② systemic and mucosal compartmentalization of antibody protection	Systemic antibodies can support lower-airway clearance, but strong circulating antibody responses do not necessarily eliminate upper-airway carriage or ensure mucosal protection	Pediatric MPP cohorts and experimental *M. pneumoniae* studies	(Meyer Sauteur et al., 2018; Meyer Sauteur et al., 2019; Meyer Sauteur et al., 2020; Lai et al., 2022)

ACOD1, aconitate decarboxylase 1; ATP, adenosine triphosphate; IRG1, immune-responsive gene 1; MPP, Mycoplasma pneumoniae pneumonia; NET, neutrophil extracellular trap; NF-κB, nuclear factor-κB; NLRP3, NOD-like receptor family pyrin domain-containing 3; RMPP, refractory Mycoplasma pneumoniae pneumonia; ROS, reactive oxygen species; TLR, Toll-like receptor; Treg, regulatory T cell.

## 5 Core carbon-metabolic circuits in inflammation and persistence

### 5.1 Glycolytic flux determines the quality, not simply the magnitude, of inflammation

Inflammatory activation commonly increases glycolysis, but its biological effect depends on how glucose-derived carbon is distributed among ATP production, lactate generation, the pentose phosphate pathway and biosynthesis. Glycolysis is therefore not intrinsically pathological; its significance depends on cell type, carbon allocation and whether metabolic activation declines with pathogen burden. M. pneumoniae-induced epithelial glycolysis supports both ATP-dependent inflammatory signalling and bacterial elimination (He et al., 2024).

Macrophage glycolysis is more directly linked to inflammatory transcription. Toll-like receptor activation remodels the tricarboxylic acid cycle, allowing succinate accumulation, hypoxia-inducible factor 1α stabilization and IL-1β production (Tannahill et al., 2013). Pyruvate kinase M2 further supports hypoxia-inducible factor 1α-dependent inflammatory gene expression, whereas its tetramerization reduces glycolysis and succinate accumulation (Palsson-McDermott et al., 2015). Although these mechanisms have been established mainly in lipopolysaccharide-stimulated macrophages, they illustrate how persistent receptor signalling and tissue hypoxia could reinforce inflammatory metabolism in RMPP even as pathogen burden declines.

The pentose phosphate pathway determines whether glucose metabolism supports oxidative killing, antioxidant defence or biosynthesis. NADPH supplies both the phagocyte oxidative burst and glutathione- and thioredoxin-dependent antioxidant systems. Neutrophils may therefore retain sufficient glycolysis for migration, degranulation and NET formation while directing inadequate reducing capacity towards bacterial killing. This uncoupling is consistent with the M. pneumoniae-induced itaconate phenotype, in which inflammatory-cell persistence coexists with suppression of mitochondrial reactive oxygen species-dependent killing (Wang C. et al., 2024).

Lactate is similarly context dependent. Its extracellular concentration reflects production, transport, tissue perfusion and cellular uptake rather than glycolytic flux alone. Lactic acid can suppress T-cell proliferation, cytokine production and cytotoxic function (Fischer et al., 2007), although these observations cannot be transferred directly from tumour microenvironments to RMPP. Nevertheless, lactate and proton accumulation in hypoxic consolidation could sustain innate inflammatory programmes while constraining lymphocyte function and epithelial repair.

If a glycolysis-dominant state is to be tested prospectively in RMPP, it should be operationalized through relationships among glucose utilization, lactate production, pentose-phosphate activity, cellular redox function and pathogen clearance rather than inferred from an isolated circulating metabolite. Plasma lactate and lactate dehydrogenase cannot resolve these processes; lactate dehydrogenase is associated with RMPP severity but primarily reflects tissue injury and cell turnover rather than cell-specific glycolytic flux (Zhang et al., 2016; Tong et al., 2022). Broad glycolytic inhibition may reduce cytokine production but could also impair epithelial antimicrobial defence, barrier maintenance and tissue repair, as well as neutrophil and lymphocyte function. More selective intervention would require identification of the stage at which glycolysis becomes uncoupled from pathogen control.

### 5.2 Mitochondria convert metabolic stress into antimicrobial and inflammatory signals

Mitochondria remain central signalling organelles even in cells that depend predominantly on glycolysis. They regulate reactive oxygen species, tricarboxylic acid cycle metabolites, apoptosis, mitochondrial DNA release and inflammasome activation, thereby influencing whether immune activation produces bacterial clearance or tissue injury.

Controlled mitochondrial reactive oxygen species contribute to antimicrobial defence. Toll-like receptor signalling can reposition mitochondria towards phagosomes and enhance macrophage bactericidal activity (West et al., 2011). Conversely, itaconate-mediated suppression of mitochondrial reactive oxygen species reduces neutrophil killing of M. pneumoniae (Wang C. et al., 2024). Mitochondrial oxidants should therefore not be interpreted uniformly as damage markers; insufficient production may compromise killing, whereas excessive or spatially uncontrolled production can injure host tissue.

Persistent mitochondrial injury promotes inflammasome signalling. Impaired removal of damaged mitochondria can activate NLRP3 (Zhou et al., 2011), while autophagy-deficient macrophages accumulate dysfunctional mitochondria and release mitochondrial DNA, increasing NLRP3-dependent IL-1β and IL-18 production (Nakahira et al., 2011). Toll-like receptor signalling can also stimulate new mitochondrial DNA synthesis, after which oxidized mitochondrial DNA directly activates NLRP3 (Zhong et al., 2018). Mitochondria thus contribute both to inflammasome priming and to the signals required for cytokine maturation.

Relevant components of this circuit are present during M. pneumoniae infection. CARDS toxin modifies NLRP3, whereas bacterial lipids connect Toll-like receptor 4 signalling with autophagy and inflammasome activation (Bose et al., 2014; Luo et al., 2021). Infection also induces epithelial reactive oxygen species and DNA damage (Sun et al., 2008). These findings do not establish a complete mitochondrial DNA-NLRP3 pathway in paediatric RMPP, but they support a model in which epithelial injury, extracellular ATP, ion flux and mitochondrial oxidants sustain inflammation after bacterial replication has partly declined.

Tricarboxylic acid cycle remodelling further converts mitochondrial metabolism into inflammatory signalling. Succinate stabilizes hypoxia-inducible factor 1α and enhances IL-1β expression (Tannahill et al., 2013), while citrate export provides acetyl coenzyme A for lipid synthesis and inflammatory mediator production. The inflammatory tricarboxylic acid cycle should therefore be viewed as a source of regulatory metabolites rather than simply as an interrupted energy-producing pathway.

Itaconate occupies a cell-dependent position in this network. In experimental macrophage systems, ACOD1-derived itaconate can inhibit succinate dehydrogenase and remodel inflammatory metabolism (Lampropoulou et al., 2016). Electrophilic itaconate derivatives can activate NRF2 through KEAP1 modification, although these derivatives do not fully reproduce the signalling properties of endogenous itaconate (Mills et al., 2018). Importantly, studies in human macrophages show that endogenously produced itaconate restrains innate cytokine production, reinforcing the view that its biological effects are strongly dependent on cellular context (Bourner et al., 2024). By contrast, experimental M. pneumoniae infection indicates that excessive neutrophil itaconate can suppress mitochondrial reactive oxygen species, impair bacterial killing and delay apoptosis (Wang C. et al., 2024). These observations support cell-context dependence rather than a uniformly beneficial or pathogenic role for itaconate. Whether comparable quantitative alterations in ACOD1 activity and itaconate flux occur in paediatric RMPP neutrophils remains unknown.

Systemic biomarkers cannot adequately represent these pulmonary processes because consolidated lung regions contain oxygen, nutrient and oxidant gradients absent from blood. Paired blood and bronchoalveolar sampling, cell separation and functional mitochondrial assays are required to distinguish local mechanisms from systemic consequences. Taken together, these observations support a hypothesis of compartment- and dose-dependent redox imbalance: insufficient mitochondrial oxidant generation may impair antimicrobial killing, whereas excessive oxidant stress may promote tissue injury and NLRP3 activation; altered itaconate signalling may further modify this balance in a cell-specific manner.

### 5.3 AMPK-mTOR-autophagy coupling determines whether metabolic stress resolves or propagates inflammation

AMP-activated protein kinase and mechanistic target of rapamycin complex 1 coordinate the transition between anabolic immune activation and catabolic repair. Mechanistic target of rapamycin complex 1 supports translation, glycolysis and immune-cell expansion, whereas AMP-activated protein kinase promotes mitochondrial quality control and autophagy during energy stress. Effective defence may therefore require an early effector phase followed by timely activation of restorative programmes rather than persistent dominance of either pathway.

Autophagy links energy regulation to inflammasome control by recycling substrates and removing damaged organelles. Loss of ATG16L1 increases caspase-1 activation and IL-1β production, demonstrating that autophagy can restrain inflammatory signalling (Saitoh et al., 2008). Inadequate mitochondrial clearance likewise permits accumulation of reactive oxygen species and mitochondrial DNA, strengthening NLRP3 activation (Nakahira et al., 2011; Zhou et al., 2011).

Its role in M. pneumoniae infection is nevertheless context dependent. Cytadherence induces Toll-like receptor 4- and autophagy-dependent epithelial inflammation (Shimizu et al., 2014), whereas bacterial lipids promote reciprocal activation of Toll-like receptor 4, autophagy and NLRP3 in macrophages (Luo et al., 2021). Autophagy may therefore either limit inflammation by removing damaged components or propagate it through continued pathogen-sensing pathways.

This apparent contradiction partly reflects the distinction between autophagy induction and completed autophagic flux. Increased autophagosome formation or LC3-II abundance does not prove successful lysosomal degradation. Persistent microbial contact, hypoxia and oxidative stress may induce autophagy while impairing lysosomal acidification or cargo clearance, allowing damaged mitochondria and inflammatory substrates to accumulate. Functional studies should therefore assess flux using lysosomal blockade, tandem fluorescent reporters or cargo turnover rather than static autophagy markers alone.

AMP-activated protein kinase can promote antimicrobial autophagy through PPARGC1A-associated mitochondrial and lysosomal programmes in experimental bacterial infection (Yang et al., 2014). Because M. pneumoniae is predominantly extracellular and epithelial-attached, the relevant benefit in RMPP may involve mitochondrial quality control, inflammasome restraint and epithelial preservation rather than direct sequestration of the organism. Mechanistic target of rapamycin inhibition is similarly stage dependent: it may reduce glycolytic cytokine production and promote autophagy but also impair lymphocyte expansion, antibody responses and tissue regeneration.

Failure to remove damaged mitochondria may allow persistent NLRP3 activation, cytokine maturation and membrane injury to become self-sustaining after the initiating microbial stimulus diminishes. The AMPK-mTOR-autophagy-inflammasome network should therefore be regarded as a decision architecture rather than a uniformly protective or pathological pathway. RMPP may emerge when anabolic inflammation persists despite energy stress, autophagy is induced but incomplete, and unresolved mitochondrial damage is converted into continued tissue injury. Until these states can be defined temporally and by cell type, therapeutic manipulation of this axis should remain mechanism-driven rather than empirical.

## 6 Lipid and amino-acid metabolism in RMPP

### 6.1 Lipid remodelling at the interface of host–pathogen interactions, membrane stress and tissue injury

Lipids are not merely by-products of immune activation. In the infected airway, phospholipids maintain epithelial membranes and surfactant, sphingolipids regulate stress signalling and cell death, neutral lipids support intracellular storage, and polyunsaturated fatty acids generate both inflammatory and pro-resolving mediators. Because M. pneumoniae has limited biosynthetic capacity and depends on host-derived lipids, infection may remodel the host lipidome through both immune-cell activation and pathogen-driven nutrient acquisition (Waites et al., 2017).

Direct evidence of local lipid remodelling has emerged from bronchoalveolar lavage fluid lipidomics. Thirteen lipid species differed between RMPP and non-refractory MPP, with DG(34:4e), PC(36:5) and SM(d38:3) showing discriminatory performance and correlations with inflammatory, tissue-injury and coagulation markers (Ma et al., 2025). However, these cross-sectional findings cannot determine whether the altered lipids originate from epithelial membranes, surfactant disruption, infiltrating leukocytes or damaged tissue. Their main contribution is therefore to demonstrate compartment-specific lipid remodelling rather than establish individual lipid species as causal biomarkers.

Peripheral metabolomic studies support broader metabolic disruption but cannot substitute for airway measurements. Plasma profiling identified changes in lipid and energy metabolism, including glycerophospholipid-related pathways (Li et al., 2022), while integrated plasma lipidomics and urine metabolomics demonstrated coordinated alterations associated with disease severity (Chen H. et al., 2024). These compartments are not interchangeable: circulating profiles are influenced by systemic inflammation, nutritional state and treatment, whereas lavage fluid more closely reflects the lung but remains sensitive to dilution and sampling procedures (Li et al., 2022; Chen H. et al., 2024).

Macrophage lipid handling may connect microbial metabolism to host inflammation. M. pneumoniae glycerol-3-phosphate dehydrogenase promotes oxidative stress, macrophage lipid uptake and a foam-cell-like phenotype, although its relevance to paediatric RMPP remains unconfirmed (Yamamoto et al., 2025). Lipid droplets may similarly sequester toxic fatty acids while organizing antimicrobial proteins and lipid-mediator synthesis (Bosch et al., 2020). In activated macrophages, however, triacylglycerol synthesis and lipid-droplet formation can enhance inflammatory activity even when fatty-acid oxidation is reduced (Castoldi et al., 2020). One hypothesis is that some forms of severe MPP involve altered lipid allocation rather than a simple increase in lipid synthesis. However, the relative contributions of pathogen lipid acquisition, host inflammatory metabolism, oxidative modification and secondary tissue injury remain unresolved.

This imbalance may extend to oxylipin metabolism. Urine metabolomics identified 16(R)-hydroxyeicosatetraenoic acid as part of a severe-MPP biomarker panel, but its enzymatic source and functional direction remain uncertain. Conversely, specialized pro-resolving mediators can increase bacterial phagocytosis, accelerate resolution and reduce antibiotic requirements in experimental infection. Such mediators are conceptually attractive because they may limit tissue injury without broadly suppressing antimicrobial defence, but defective pro-resolving mediator production has not been demonstrated in paediatric RMPP (Chiang et al., 2012; Li L. et al., 2025). Current evidence therefore supports compositional and compartmental lipid remodelling, while causal interpretation requires targeted lipid-mediator profiling and cell-specific validation.

### 6.2 Amino-acid metabolism couples inflammatory activation to immune-cell fate

Among amino-acid pathways, tryptophan metabolism currently has the strongest replicated clinical evidence within paediatric MPP, but the available data do not establish RMPP specificity. Paediatric plasma multi-omics has demonstrated activation of the kynurenine pathway together with suppression of several indole-related metabolites, with both changes associated with inflammatory severity (Tang et al., 2025). Urine metabolomics has independently identified kynurenine and 3-hydroxyanthranilic acid within a panel distinguishing severe from general MPP (Li L. et al., 2025). This convergence supports tryptophan reprogramming as a reproducible correlate of severe MPP rather than an established mechanism specific to refractoriness. This distinction is important because activation of the kynurenine pathway is also observed in other severe infectious syndromes. Increased kynurenine pathway activity has been associated with inflammatory burden and adverse outcomes in community-acquired pneumonia (Meier et al., 2017), while an increased plasma kynurenine-to-tryptophan ratio has also been demonstrated in sepsis and associated with systemic immune and vascular dysfunction (Darcy et al., 2011). The pathway may therefore represent a broader interferon-driven response to severe infection rather than an RMPP-specific metabolic signature. Its ability to distinguish RMPP from other bacterial or viral pneumonias of comparable inflammatory severity remains unknown and should be tested using severity-matched etiological control groups.

Interferon-induced indoleamine 2,3-dioxygenase 1 may provide the mechanistic link between inflammation and tryptophan catabolism. Tryptophan depletion can restrict lymphocyte proliferation, whereas kynurenine metabolites regulate T cells, macrophages and epithelial cells through amino-acid stress and aryl hydrocarbon receptor pathways. The prominent interferon-γ-producing CD4^+^ response in MPP provides a plausible stimulus for this pathway (Pánisová et al., 2021). Sustained activation could therefore restrain excessive inflammation while simultaneously weakening protective adaptive immunity. The concurrent reduction in microbiota-derived indoles may further impair epithelial and regulatory signalling, although it remains unclear whether this precedes severe disease or results from fever, diet and antibiotic exposure (Tang et al., 2025).

Arginine metabolism represents a less established candidate axis. Severe-MPP urine profiles show enrichment of arginine-biosynthesis pathways (Li L. et al., 2025), but these data cannot distinguish nitric oxide synthase from arginase activity. The former may support antimicrobial defence but increase nitrosative injury, whereas the latter can restrict nitric oxide production while promoting polyamine synthesis and tissue remodelling. Without direct measurements of arginine availability and pathway flux in pulmonary immune cells, arginine metabolism should be regarded as a hypothesis generated by metabolomics rather than a confirmed mechanism of refractoriness.

Glutamine similarly supports tricarboxylic acid cycle replenishment, nucleotide synthesis and glutathione production, thereby linking immune-cell proliferation to redox control. Experimental modulation of glutamate-dependent metabolism changes α-ketoglutarate availability and epigenetic regulation of the FOXP3 locus, shifting the Th17-regulatory T-cell balance (Xu et al., 2017). This provides a mechanistic connection to the Th17-Treg abnormalities reported in RMPP (Guo et al., 2016; Yang et al., 2017), but direct evidence of altered glutamine flux in affected children is lacking. Overall, tryptophan reprogramming has replicated clinical support, whereas arginine and glutamine pathways remain priorities for cell-specific and functional validation rather than established therapeutic targets.

### 6.3 Redox metabolism may shape how lipid and amino-acid stress is converted into cell death

Lipid and amino-acid metabolism converge on redox control. Polyunsaturated phospholipids provide oxidation substrates, amino acids support glutathione synthesis, NADPH sustains both oxidant production and antioxidant recycling, and iron promotes lipid-radical propagation. M. pneumoniae adds directly to this pressure: pathogen-derived superoxide and hydrogen peroxide can damage host cells (Almagor et al., 1984), while infection also induces epithelial reactive oxygen species, mitochondrial stress and DNA injury (Sun et al., 2008). Neutrophils, macrophages and epithelial mitochondria provide additional oxidant sources, making tissue injury the combined product of microbial metabolism and host defence.

Oxidant exposure does not uniformly eliminate infected cells. M. pneumoniae can protect infected epithelial cells from hydrogen peroxide-induced detachment, potentially preserving its mucosal niche while neighbouring cells are lost to oxidants, proteases and nutrient deprivation (Yamamoto et al., 2019). This spatially uneven response may help explain the coexistence of persistent microbial stimulation and progressive epithelial-barrier disruption.

Different death programmes may then determine whether cellular injury resolves or amplifies inflammation. CARDS toxin and M. pneumoniae-derived lipids activate NLRP3-related pathways and promote IL-1β and IL-18 processing (Bose et al., 2014; Luo et al., 2021), but inflammasome activation alone does not prove complete pyroptotic execution. Neutrophil apoptosis is also context dependent: delayed apoptosis mediated by the itaconate pathway prolongs the survival of cells with impaired bacterial killing, thereby sustaining ineffective inflammation (Wang C. et al., 2024). By contrast, excessive epithelial apoptosis may compromise mucociliary clearance and repair.

Ferroptosis is relevant to this redox framework because it is defined by iron-dependent phospholipid peroxidation (Dixon et al., 2012). In paediatric RMPP, however, direct functional evidence of ferroptotic cell death remains lacking; ferroptosis-related transcriptional or molecular signatures should be regarded as hypothesis-generating unless accompanied by cell-specific lipid peroxidation, evidence of iron dependence and pathway-specific rescue. Etiological confounding is an additional concern. Respiratory viral co-infections, including adenovirus and influenza, can substantially alter airway inflammatory and metabolic programmes in paediatric MPP (Li Z. et al., 2025). Such co-infections may therefore contribute to ferroptosis-related molecular signatures independently of the mechanism responsible for refractoriness. Functional ferroptosis has also been demonstrated in influenza-associated lung epithelial injury, including iron dysregulation, phospholipid peroxidation and rescue by ferrostatin-1 (Zheng et al., 2025). Future RMPP studies should consequently perform systematic respiratory co-pathogen testing and require evidence of iron-dependent lipid peroxidation together with pathway-specific rescue before attributing tissue injury specifically to M. pneumoniae-induced ferroptosis.

## 7 Multi-omics evidence, the gut-lung axis and biomarker discovery

### 7.1 Multi-omics should resolve disease architecture, not merely expand the biomarker catalogue

Multi-omics is valuable in RMPP because heterogeneous immune activation, variable pathogen persistence and divergent pulmonary outcomes are unlikely to be explained by a single marker. Its value, however, depends on whether molecular layers converge on coherent programmes linked to defined cells, anatomical compartments and disease trajectories. Single-cell analysis of bronchoalveolar lavage fluid has identified coordinated remodelling of epithelial, myeloid and lymphoid populations, including severity-associated macrophage states, enhanced neutrophil programmes and altered intercellular communication (Shen et al., 2024). These findings support cellular heterogeneity, but transcriptional states do not directly establish metabolic flux.

Metabolomic studies provide complementary biochemical evidence. Plasma studies demonstrate alterations in tryptophan, lipid and energy metabolism, with activation of the kynurenine pathway and suppression of indole-related metabolites associated with inflammatory severity (Li et al., 2022; Chen H. et al., 2024; Tang et al., 2025). Urine profiles implicate kynurenine-related, arginine-related and lipid-oxidation pathways (Li L. et al., 2025), whereas bronchoalveolar lavage-fluid lipidomics identifies local changes in phosphatidylcholine, sphingomyelin and diacylglycerol species in RMPP (Ma et al., 2025). Together, these findings support metabolic remodelling across systemic and pulmonary compartments, but differences among plasma, urine and lavage fluid reflect distinct biological sources and sampling constraints.

Cross-sectional sampling limits causal interpretation. Samples obtained after prolonged fever, corticosteroid exposure, viral co-infection or established lung injury may capture treatment effects and tissue damage rather than the mechanisms that initiated refractoriness. Longitudinal studies should therefore distinguish early infection, progression and resolution, and cross-omics associations should be linked to functional perturbation before they are interpreted as causal pathways.

Airway multi-omics illustrates both the value and limitations of this approach. Viral co-infection has been associated with extensive metabolic remodelling, depletion of Lactobacillus, increased CCL-family chemokines and monocyte-associated inflammation (Li Z. et al., 2025). However, these findings distinguish co-infected from singly infected MPP rather than define an RMPP-specific programme. Clinically informative analysis should integrate the dominant pulmonary cell state, the metabolic process supporting that state, residual pathogen burden and the anatomical pattern of injury. Current studies mainly establish interacting cellular and metabolic programmes; the next priority is to determine which programmes precede deterioration and which reflect established injury.

### 7.2 The airway and intestinal microbiota shape the metabolic context of disease

Respiratory microbiome studies demonstrate altered community composition and interactions between M. pneumoniae abundance and the wider airway ecosystem in paediatric MPP (Wang et al., 2023). In macrolide-resistant MPP, prolonged fever and the need for doxycycline were associated with lower respiratory microbial diversity and distinct oropharyngeal communities linked to amino-acid, fatty-acid and glycerophospholipid metabolites (Liao et al., 2025). These associations connect microbial ecology with host metabolism but do not establish that individual taxa are protective or pathogenic, because age, sampling site, antimicrobial exposure, airway obstruction and treatment decisions all influence relative abundance.

Co-infection further reorganizes this ecosystem. Adenovirus and influenza A co-infection produce distinct metabolic and inflammatory patterns, and adenovirus-associated loss of Lactobacillus accompanies monocyte-related chemokine activation (Li Z. et al., 2025). Intestinal studies likewise report gut microbial and serum-metabolite alterations in paediatric MPP and differences between children with and without wheezing (Jiang et al., 2022; Wang S. et al., 2024). However, antibiotics, diet, fever and hospitalization remain major confounders, and intestinal dysbiosis has not been shown to cause refractory or post-infectious airway disease.

Short-chain fatty acids, indole derivatives and bile-acid metabolites provide plausible mediators of gut-lung communication. Reduced indole-related metabolites in paediatric MPP and *in vitro* suppression of M. pneumoniae-induced macrophage inflammation by acetate, propionate and butyrate support this possibility (Wen et al., 2021; Tang et al., 2025). Nevertheless, these findings do not establish metabolite deficiency in RMPP or justify supplementation. Prospective pretreatment sampling, absolute microbial quantification, metagenomic functional profiling and direct metabolite measurement are required to distinguish causal microbial functions from compositional changes. The microbiota should therefore be viewed as a metabolic boundary condition that can modify host responses but is itself remodelled by infection, treatment and airway physiology.

### 7.3 Biomarkers must progress from predicting severity to defining testable biological states

Most RMPP biomarkers were developed to identify children likely to fail macrolide therapy, but risk prediction does not necessarily reveal mechanism. Lactate dehydrogenase, ferritin and IL-18 can help distinguish refractory from uncomplicated disease (Lu et al., 2015; Choi et al., 2019), yet they primarily reflect inflammation or tissue injury and cannot separate persistent infection, neutrophil dysfunction, epithelial stress or anatomical obstruction.

Clinical prediction models improve discrimination by combining demographic, laboratory and imaging variables. Paediatric scores incorporating age, fever duration and inflammatory or biochemical indices identify children at increased RMPP risk, while an XGBoost model highlighted fever duration, previous macrolide therapy, severe pneumonia, lactate dehydrogenase, neutrophil-to-lymphocyte ratio, alanine aminotransferase, peak temperature and extensive consolidation (Bi et al., 2021; Jiang et al., 2025). However, several of these variables are already embedded in the clinical recognition of refractoriness, creating temporal leakage and limiting biological interpretation and transportability.

Transcriptomic classifiers offer a route beyond conventional severity markers. A multinational paediatric study identified a host blood transcriptional response that distinguished MPP from other infectious conditions (Viz-Lasheras et al., 2025), and a peripheral-blood mononuclear-cell four-gene model differentiated healthy children, general MPP and RMPP (Wu et al., 2026). These approaches move classification towards host-response states, but etiological or diagnostic discrimination does not establish why progression occurs. Prospective external validation and linkage of classifier genes to defined cellular functions and treatment responses are still required.

Candidate biological states should be evaluated by whether they add mechanistic information beyond conventional severity markers and remain informative at a prespecified treatment-decision point. Longitudinal integration of pathogen burden, cell-specific function, tissue injury and anatomical progression should therefore precede any claim that a molecular configuration represents a clinically actionable endotype. Representative human multi-omics and biomarker studies relevant to paediatric MPP and RMPP are summarized in **Table 2**.

**Table 2:** Representative human multi-omics and biomarker evidence in paediatric Mycoplasma pneumoniae pneumonia and refractory disease.

Approach	Main finding	Potential clinical value	Principal limitation	Reference
Bronchoalveolar single-cell transcriptomics	Identified coordinated remodelling of epithelial, macrophage, neutrophil and lymphocyte populations associated with disease severity	Discovery of cell-specific host-response states for future endotype testing	Cross-sectional transcriptional states do not directly demonstrate metabolic flux or causal function	(Shen et al., 2024)
Plasma multi-omics and metabolomics	Demonstrated activation of the kynurenine pathway, suppression of indole-related metabolites, and alterations in amino-acid, lipid and energy metabolism	Identification of systemic metabolic pathways associated with inflammatory severity	Cellular sources and causal direction remain uncertain; findings may be influenced by treatment, diet and systemic inflammation	(Li et al., 2022; Chen H. et al., 2024; Tang et al., 2025)
Bronchoalveolar lavage-fluid lipidomics	Identified 13 differential lipid species between RMPP and non-refractory MPP; DG(34:4e), PC(36:5) and SM(d38:3) showed discriminatory potential	Provides a local pulmonary lipid signature associated with inflammation, tissue injury and coagulation	Cross-sectional design, lavage dilution and uncertain cellular origin limit causal interpretation	(Ma et al., 2025)
Urine metabolomics combined with machine learning	Identified kynurenine-related, arginine-related and lipid-oxidation pathways that differentiated severe from general MPP	Non-invasive candidate panel for severity stratification	Evaluated severe MPP rather than a prospectively defined RMPP endotype; external validation is required	(Li L. et al., 2025)
Airway microbiome and integrated multi-omics	Viral co-infection and altered respiratory microbial communities were associated with distinct inflammatory and metabolic patterns; lower microbial diversity was associated with more severe macrolide-resistant disease	May support ecological and co-infection-based stratification	Antibiotic exposure, relative-abundance analysis and reverse causation limit mechanistic interpretation	(Wang et al., 2023; Liao et al., 2025; Li Z. et al., 2025)
Conventional circulating biomarkers	LDH, ferritin and IL-18 were associated with refractory disease and may improve early risk recognition	Widely accessible clinical risk stratification	These markers reflect inflammation or tissue injury but do not distinguish persistent infection, immune dysfunction or airway obstruction	(Lu et al., 2015; Choi et al., 2019)
Clinical and machine-learning prediction models	Scores incorporating age, fever duration, laboratory indices and imaging features, as well as XGBoost-based models, identified children at increased RMPP risk	Early clinical risk prediction using routinely available variables	Potential temporal leakage, single-centre overfitting and limited external validation	(Bi et al., 2021; Jiang et al., 2025)
Host blood transcriptomic classifiers	Host-response signatures distinguished MPP from other infections, and a four-gene model differentiated healthy children, general MPP and RMPP	Moves classification from nonspecific severity markers toward host-state assessment	Prospective external validation and evidence of differential treatment response are lacking	(Viz-Lasheras et al., 2025; Wu et al., 2026)

DG, diacylglycerol; IL, interleukin; LDH, lactate dehydrogenase; MPP, Mycoplasma pneumoniae pneumonia; PC, phosphatidylcholine; RMPP, refractory Mycoplasma pneumoniae pneumonia; SM, sphingomyelin; XGBoost, extreme gradient boosting.

## 8 Therapeutic reframing through candidate biological states

### 8.1 Existing therapies target distinct layers of the host-pathogen system

RMPP treatment commonly progresses from macrolides to alternative antibiotics, corticosteroids and bronchoscopy, but these interventions address different components of disease persistence. Antibiotics reduce viable pathogen burden, corticosteroids suppress disproportionate inflammation, and bronchoscopy removes mucus plugs or casts that sustain obstruction. They are therefore complementary rather than interchangeable.

Persistent fever after macrolide treatment may reflect resistance, delayed active therapy, toxin-mediated injury, airway obstruction or inflammation that has become partly independent of pathogen burden. Doxycycline, levofloxacin and corticosteroids can all shorten fever duration in macrolide-unresponsive MPP, although treatment selection is confounded by age, severity and local practice (Ha et al., 2018). Defervescence consequently demonstrates clinical response but does not identify which pathogenic process has been corrected.

Tetracyclines provide a microbiologically targeted alternative in older children. Early minocycline is associated with faster defervescence and less progression from macrolide-unresponsive to refractory disease (Chen et al., 2021). Doxycycline is likewise more effective when introduced early in macrolide-resistant infection (Chen Y. et al., 2024). Real-world comparisons support both doxycycline and levofloxacin, but paediatric safety restrictions and confounding by indication prevent a universal hierarchy (Yang et al., 2025). The consistent implication is that delayed active treatment prolongs microbial stimulation and increases the opportunity for inflammation to become self-sustaining.

The challenge is identifying children with continued pathogen-dominant disease. Current prediction models frequently use fever duration, inflammatory markers and radiographic progression, which may already represent established refractoriness (Xie et al., 2022). More informative models should combine resistance genotype with quantitative pathogen burden and its trajectory after treatment.

Corticosteroids target persistent inflammation rather than bacterial replication. Meta-analyses show that adjunctive glucocorticoids shorten fever and hospitalization and reduce inflammatory indices in macrolide-refractory disease (Kim et al., 2019; Qiu et al., 2020). Prednisolone and methylprednisolone can also produce rapid improvement in selected children with progressive pneumonia (Luo et al., 2014). However, heterogeneous definitions, doses and timing limit regimen selection, and most studies emphasize defervescence rather than stable radiographic recovery or long-term pulmonary function.

Glucocorticoids interrupt epithelial-myeloid amplification by suppressing inflammatory transcription, cytokines and leukocyte trafficking, but they do not selectively restore bacterial killing. Children with elevated lactate dehydrogenase, ferritin or related inflammatory markers may improve when host-mediated injury dominates (Lu et al., 2015; Choi et al., 2019). Persistent fever despite corticosteroids may instead indicate active infection, necrosis, mucus obstruction or glucocorticoid-insensitive inflammation; it should not be interpreted simply as greater activity of the same pathway.

Bronchoscopy addresses established anatomical disease. Mucus plugs and casts restrict ventilation and retain extracellular DNA, proteases, fibrin and microbial material. Timely lavage and cast removal can improve clinical and radiographic outcomes in MPP-associated plastic bronchitis (Zhong et al., 2021), but they do not correct the epithelial, coagulation or neutrophil programmes producing obstruction. Recurrence remains possible while mucin production, NET accumulation and local inflammation persist.

Current treatment can therefore be organized around three objectives: removing microbial stimulation, suppressing disproportionate inflammation and relieving anatomical obstruction. Combination therapy is appropriate when these processes coexist, but escalation should reflect the dominant biological state rather than severity alone.

### 8.2 Host-directed therapy should restore immune efficiency, not simply suppress inflammation

Potential host-directed targets include NLRP3, extracellular ATP, neutrophil itaconate, NETs, glycolysis, mitochondrial stress and redox regulation. Because each contributes to both defence and injury, treatment should improve the ratio of pathogen clearance to collateral damage rather than simply lower inflammatory markers.

NLRP3 is a plausible convergent target. CARDS toxin activates NLRP3 directly, while M. pneumoniae lipids connect Toll-like receptor signalling, autophagy and inflammasome activation (Bose et al., 2014; Luo et al., 2021). IL-1β and IL-18 are also associated with severe or refractory disease (Zhang et al., 2016; Choi et al., 2019). The inhibitor MCC950 demonstrates that NLRP3 can be pharmacologically separated from upstream innate signalling in experimental models (Coll et al., 2015). However, NLRP3 contributes to antimicrobial defence, and no clinical evidence supports its inhibition in paediatric RMPP. Intervention would require evidence that pulmonary inflammasome activity remains excessive after microbial control.

Epithelial purinergic signalling has similar temporal dependence. Infection-induced glycolysis promotes extracellular ATP release and P2X7-associated inflammation but also supports bacterial elimination (He et al., 2024). Early inhibition might impair defence, whereas later inhibition could be beneficial if injured cells continue releasing ATP after pathogen burden declines. No available biomarker reliably distinguishes these phases.

The neutrophil ACOD1-itaconate pathway should currently be regarded as a mechanistic candidate rather than a therapeutic target. Experimental suppression of neutrophil itaconate improves bacterial killing and accelerates apoptosis in M. pneumoniae-infected mice (Wang C. et al., 2024), but this intervention has not been validated in paediatric RMPP. Moreover, endogenous itaconate restrains inflammatory signalling in human macrophages (Bourner et al., 2024), raising the possibility that systemic ACOD1 inhibition could improve antimicrobial function in one cellular compartment while removing protective regulation in another. Therapeutic development would therefore require confirmation of pathway activity in paediatric pulmonary neutrophils, cell-selective target engagement and evidence that macrophage-mediated inflammatory control and resolution are preserved. At present, no clinical recommendation can be made for ACOD1 or itaconate modulation in RMPP.

NET-directed interventions may be relevant to mucus-dominant disease. NETs contribute extracellular DNA, histones and proteases to casts, while M. pneumoniae can degrade their DNA scaffold and partially escape killing (Yamamoto et al., 2017; Huang et al., 2025). PAD4 inhibition, histone neutralization or local DNase is mechanistically plausible, but effectiveness will depend on cast composition because mucin, fibrin and active proteases may remain after DNA degradation.

Broad metabolic suppression is less attractive. Glycolytic inhibition may reduce cytokines but impair epithelial bacterial elimination, neutrophil activity and lymphocyte expansion (He et al., 2024). Generic antioxidants could reduce tissue injury while weakening reactive oxygen species-dependent killing (West et al., 2011; Wang C. et al., 2024). Likewise, mTOR inhibition or AMP-activated protein kinase activation may promote repair at one stage but restrict immune expansion or epithelial recovery at another. Treatment should therefore target a pathological pathway configuration rather than suppress metabolism globally.

Specialized pro-resolving mediators represent an alternative strategy by promoting neutrophil clearance, macrophage efferocytosis and tissue restoration while preserving bacterial phagocytosis in experimental infection (Chiang et al., 2012). This is attractive when delayed neutrophil apoptosis and failed resolution dominate, but deficient mediator production and therapeutic benefit have not been demonstrated in paediatric RMPP. The kynurenine-aryl hydrocarbon receptor axis is similarly context-dependent: increased tryptophan catabolism may restrain inflammation but impair adaptive defence, whereas reduced microbiota-derived indoles may weaken epithelial and regulatory functions (Tang et al., 2025). Causality must be separated from the effects of antibiotics, diet and systemic inflammation before intervention.

The most compelling targets are therefore those that restore coordination between killing and resolution, particularly neutrophil itaconate, persistent NETs and defective pro-resolving signalling. Even these require confirmation of pathway activity, timing and cell-specific effects.

### 8.3 Precision treatment requires state-based rather than severity-based trials

Current trials generally enrol children based on persistent fever, radiographic progression or macrolide failure. These criteria combine biologically different processes and may dilute treatment effects. The states proposed below should therefore be regarded as candidate research constructs rather than validated clinical endotypes. No assay-specific numerical thresholds have yet been validated for paediatric RMPP, and fixed cut-offs should not be inferred from current case–control studies. Initial operationalization should instead rely on prespecified serial measurements, comparison with appropriate non-refractory MPP controls and concordance across microbial, cellular-functional and anatomical domains. Candidate measurements are summarized in **Table 3**.

**Table 3:** Provisional operational framework for candidate biological states in paediatric RMPP.

Candidate state	Operational definition for research	Candidate measurements	Dynamic interpretation	Evidence
Pathogen-persistent	Persistent microbial signal despite verified exposure to an active antimicrobial	Serial quantitative lower-airway *M. pneumoniae* PCR/ddPCR; 23S rRNA resistance genotype; antimicrobial exposure; culture where feasible	Failure of pathogen burden to decline is more informative than a single positive PCR	(Chen et al., 2021; Chen Y. et al., 2024; Wang YS. et al., 2024; Yang et al., 2025)
Host-inflammatory	Declining pathogen burden with persistent or increasing inflammatory/tissue-injury activity	Serial CRP, ferritin, IL-18, LDH; airway cytokines when clinically obtained	Discordance between microbial decline and persistent inflammatory injury supports a host-dominant state	(Luo et al., 2014; Lu et al., 2015; Choi et al., 2019; Kim et al., 2019; Qiu et al., 2020)
Neutrophil-inefficient	Neutrophil accumulation accompanied by impaired antimicrobial function and persistent microbial stimulation	Blood/BALF neutrophil ACOD1; targeted LC-MS/MS itaconate; mitochondrial ROS/oxidative burst; ex vivo killing; apoptosis	Neutrophilia alone is insufficient; functional impairment must coexist with persistent pathogen signal	(Wang C. et al., 2024)
NET–mucus/obstructive	Anatomical obstruction accompanied by NET-rich airway material	CT/bronchoscopy; extracellular DNA; MPO–DNA complexes; citrullinated histone H3; mucin/fibrin composition	Persistent obstruction despite falling systemic inflammatory markers suggests increasing anatomical dominance	(Yamamoto et al., 2017; Zhong et al., 2021; Huang et al., 2025)

A pathogen-persistent state would include high or non-declining bacterial burden, documented resistance or delayed active antimicrobial exposure. Early doxycycline, minocycline or another effective antibiotic would be prioritized, consistent with evidence that timely tetracycline treatment improves macrolide-unresponsive or resistant disease (Chen et al., 2021; Chen Y. et al., 2024; Yang et al., 2025). Prediction models should identify persistent microbial activity rather than reconstruct established refractoriness from fever and radiographic deterioration (Xie et al., 2022).

A host-inflammatory state would combine declining pathogen burden with persistent inflammatory and tissue-injury signals. This subgroup provides the strongest rationale for corticosteroids, which shorten fever and hospitalization but have mainly been evaluated through short-term outcomes (Luo et al., 2014; Kim et al., 2019; Qiu et al., 2020). Lactate dehydrogenase and ferritin may indicate inflammatory severity (Lu et al., 2015; Choi et al., 2019), but whether they predict corticosteroid benefit rather than refractoriness alone remains unknown.

A neutrophil-inefficient state would combine airway neutrophilia with impaired killing, increased itaconate or delayed apoptosis and may respond poorly to either antibiotic escalation or further immunosuppression (Wang C. et al., 2024). A related NET-mucus state would include obstruction, extracellular DNA, proteases and bronchial casts. Bronchoscopy and future local NET- or mucus-directed interventions would be more appropriate than additional systemic immunosuppression (Yamamoto et al., 2017; Zhong et al., 2021; Huang et al., 2025).

These states are dynamic and may overlap. Disease can progress from pathogen-dominant infection to autonomous inflammation and then mucus-dominant obstruction. Serial measurements of pathogen burden, inflammatory activity and airway anatomy are therefore more informative than a single baseline classification.

Antibiotic trials should test whether early active treatment prevents transition to autonomous inflammation rather than compare defervescence alone (Ha et al., 2018; Chen et al., 2021; Chen Y. et al., 2024; Yang et al., 2025). Corticosteroid trials should stratify effects by pathogen-load trajectory, inflammatory state and treatment timing (Luo et al., 2014; Kim et al., 2019; Qiu et al., 2020). Bronchoscopy studies should identify responsive anatomical phenotypes and assess recurrent obstruction rather than immediate radiographic improvement alone (Zhong et al., 2021).

Outcomes must extend beyond fever and hospital stay. Relevant measures include quantitative bacterial clearance, sustained radiographic recovery, repeated bronchoscopy, recurrent obstruction, bronchiolitis obliterans, bronchiectasis and long-term pulmonary function. Host-directed trials must capture both antimicrobial control and tissue protection because improvement in one dimension may conceal deterioration in another (West et al., 2011; Chiang et al., 2012; He et al., 2024; Wang C. et al., 2024).

The immediate goal is not to replace antibiotics, corticosteroids or bronchoscopy with experimental metabolic drugs, but to deploy existing treatments according to whether progression is driven by persistent infection, autonomous inflammation, neutrophil dysfunction or anatomical obstruction. Immunometabolic endotypes will become clinically useful only when a measurable biological state predicts differential benefit from a specific intervention.

## 9 Evidence gaps and a research agenda: from associations to actionable endotypes

A major obstacle to mechanistic research in paediatric RMPP is the instability of the clinical phenotype. Current definitions generally combine persistent fever and radiographic progression after several days of macrolide therapy, but treatment duration, imaging criteria and severity thresholds vary across studies. Although evidence-based guidance has improved the diagnosis and management of paediatric Mycoplasma pneumoniae pneumonia, RMPP remains partly defined by treatment exposure and clinical judgement rather than by a stable biological state (Subspecialty Group of Respiratory, the Society of Pediatrics, Chinese Medical Association, China National Clinical Research Center of Respiratory Diseases, & Editorial Board, Chinese Journal of Pediatrics, 2025). This creates circularity because fever duration, previous macrolide use and radiographic deterioration are often used both to define RMPP and to predict it. Molecular abnormalities measured after prolonged disease may also reflect corticosteroid exposure, fasting, tissue necrosis or airway obstruction rather than mechanisms that initiated progression.

Future studies should therefore enrol children when MPP is first suspected and obtain samples before refractoriness, second-line antimicrobial therapy or immunomodulation is established. Phenotyping should incorporate four dimensions: microbial activity, including resistance genotype, quantitative pathogen burden and exposure to an active antimicrobial agent; the dominant cellular and metabolic host-response state; anatomical injury, including consolidation, mucus plugging, bronchial casts and necrosis; and disease time relative to symptom onset, treatment and objective progression. This framework would distinguish macrolide resistance, antimicrobial non-response, severe pneumonia and post-infectious airway disease, which overlap clinically but are not biologically equivalent. Evidence should also be weighted according to its proximity to paediatric RMPP, with longitudinal paediatric studies using standardized definitions given greater weight than cross-sectional cohorts, experimental models or evidence extrapolated from unrelated inflammatory diseases.

Mechanistic studies must move beyond metabolite abundance and pathway-enrichment analysis. Accumulation of a metabolite may result from increased production, reduced utilization or tissue damage, while increased expression of glycolytic genes cannot determine whether glucose-derived carbon enters lactate production, the pentose phosphate pathway or biosynthesis. Metabolomics should therefore be combined with extracellular flux analysis, stable-isotope tracing and functional immune assays (Voss et al., 2021). Labelled glucose, glutamine or fatty acids can define substrate allocation across glycolysis, the tricarboxylic acid cycle and biosynthetic pathways. Studies of activated T cells demonstrate that increased glucose uptake supports multiple metabolic fates rather than simple conversion to lactate, illustrating why nutrient uptake alone cannot define inflammatory metabolism (Ma et al., 2019).

In paediatric RMPP, short-term ex vivo analysis of freshly isolated blood or bronchoalveolar cells may be more feasible than systemic tracer studies. Neutrophils, macrophages, lymphocytes and epithelial cells should be analysed separately because bulk lavage fluid combines biologically distinct programmes. Metabolic measurements should then be linked to relevant functions: neutrophil itaconate and redox metabolism to oxidative burst, bacterial killing, apoptosis and NET formation; macrophage metabolism to phagocytosis, efferocytosis and inflammasome activity; and epithelial metabolism to barrier integrity, mucin production and extracellular ATP release.

Single-cell RNA sequencing can identify candidate cellular states but cannot directly measure metabolic activity because enzyme function is regulated beyond transcription and metabolic transcripts may be incompletely detected. Transcript-based metabolic inference therefore requires biochemical and functional validation (Artyomov and Van den Bossche, 2020). Spatial context is equally important because ventilated airways, consolidated tissue and bronchial casts differ in oxygen availability, substrate distribution and cellular composition. Spatial transcriptomic and imaging-based metabolic approaches may help localize dysfunctional programmes, although current resolution and metabolite annotation remain limited (Hartmann, 2025). Bronchial casts obtained during clinically indicated bronchoscopy may provide particularly informative material for analysing extracellular DNA, histones, proteases, fibrin, mucins, oxidized lipids and microbial components. Paired blood and airway sampling could then determine whether accessible circulating markers represent defined pulmonary mechanisms.

An actionable endotype must represent a reproducible mechanism, be measurable using a practical biomarker and predict disease course or treatment response. A molecular signature that merely distinguishes RMPP from uncomplicated MPP remains a descriptive subtype unless it changes management. Endotype development should therefore begin with a predefined clinical decision. A classifier intended to guide antimicrobial escalation must detect persistent microbial activity before prolonged treatment failure; a corticosteroid classifier must distinguish host-dominant inflammation from uncontrolled infection; and a bronchoscopy classifier must identify anatomical obstruction unlikely to resolve with systemic treatment. These decisions reflect different mechanisms and are unlikely to be addressed by one universal score.

The prediction time point must be specified, and variables measured after the intended decision must be excluded. Fever duration on day 7 cannot guide treatment on day 3, and deterioration after corticosteroid exposure cannot predict baseline corticosteroid responsiveness. Models should be transparently reported and systematically assessed for risk of bias because many current studies are small, single-centre and vulnerable to overfitting or temporal leakage (Moons et al., 2015; Wolff et al., 2019). External validation should assess calibration, clinical utility and transportability across centres with different resistance prevalence and treatment practices, rather than discrimination alone.

Endotypes are also likely to change over time as children move from pathogen-dominant infection to host-dominant inflammation and subsequently to mucus-dominant obstruction. Serial classification may therefore be more informative than a single admission measurement. Sepsis transcriptomic studies provide proof of principle that reproducible host-response states may be associated with clinical outcomes and differential corticosteroid responses, although these findings cannot be transferred directly to paediatric RMPP (Scicluna et al., 2017; Antcliffe et al., 2019).

The decisive validation study would measure a candidate classifier prospectively before treatment allocation and test whether treatment effects differ between biomarker-defined groups. Multi-omics may support discovery, but implementation should converge on a small and robust panel. **Table 3** provides candidate operational measures for these research states, but their thresholds should be derived in longitudinal cohorts and prospectively validated before treatment allocation rather than selected retrospectively for optimal case–control discrimination. A candidate neutrophil-inefficient state, for example, should require concordance between persistent microbial stimulation, neutrophil accumulation and demonstrable functional impairment rather than an isolated increase in itaconate or neutrophil count. The immediate priority is therefore a longitudinal disease map integrating pathogen burden, cell-specific function, metabolic state, anatomical injury and treatment response. Only such a framework can determine when active antimicrobial therapy, immunomodulation or bronchoscopic intervention is most appropriate.

## 10 Conclusion

Paediatric RMPP should not be regarded as a single immunometabolic disorder. Rather, the clinical phenotype may arise from different combinations of persistent microbial stimulation, altered host responses and evolving anatomical injury. Epithelial glycolysis, macrophage inflammasome activity, neutrophil ACOD1–itaconate signalling and NET accumulation, mitochondrial and redox stress, lipid remodelling and altered amino-acid metabolism provide plausible mechanisms through which antimicrobial defence and tissue injury may become uncoupled, but their contributions are likely to differ across patients, cellular compartments and disease stages. Most current human evidence remains associative and cross-sectional, whereas several mechanistic pathways are supported predominantly by experimental models. Longitudinal studies integrating quantitative pathogen trajectories, cell-resolved metabolic function, anatomical injury and treatment response are therefore required before these immunometabolic configurations can be considered validated endotypes. The immediate value of this framework is to generate testable biological hypotheses rather than to prescribe metabolism-directed therapy.
